# Potential impacts to human health from climate change: A comparative life-cycle assessment of single-use versus reusable devices flexible ureteroscopes

**DOI:** 10.1007/s00240-024-01664-2

**Published:** 2024-11-23

**Authors:** Marlene Thöne, Jan Lask, Jörg Hennenlotter, Matthias Saar, Igor Tsaur, Arnulf Stenzl, Steffen Rausch

**Affiliations:** 1 Department of Urology, University Hospital Tübingen, Hoppe-Seyler-Str. 3, D-72076 Tübingen, Germany; 2 Department of Urology, RWTH University Hospital Aachen, Aachen, Germany; 3 Faculty of Agricultural Sciences, University of Hohenheim, Stuttgart, Germany

## Abstract

Climate change poses a significant global health challenge, with medical procedures contributing substantially to CO_2_ emissions. Urology, as part of the broader healthcare sector, has begun integrating Planetary Health concepts to address this issue. While earlier studies have focused on Life Cycle Assessments (LCA) of urological procedures, these evaluations remain data-dependent, and insights into intra-hospital emissions are limited. This study introduces a methodical approach for analyzing intra-institutional processes of LCA for single-use and reusable flexible ureterorenoscopes (fURS). The LCA method was applied to assess the greenhouse gas emissions (CO_2_ equivalents, CO_2_-eq) generated across the life cycle of fURS, including production, use-phase, reprocessing, maintenance, and disposal. The study approximated the Global Warming Potential (GWP) per one-hour use and evaluated associated health impacts using the ReCiPe2016(H) method, which measures Disability-Adjusted Life Years (DALYs). Results showed that for reusable fURS, assuming 133 usages per device and maintenance after every 11th use, each application generated 1.24 kg CO_2_-eq, equivalent to 1.15E-06 DALYs. In contrast, single-use fURS generated 4.93 kg CO_2_-eq and 4.57E-06 DALYs per application. The production and reprocessing stages were identified as having the greatest environmental and health impacts. For reusable fURS, electricity required during refurbishment and use phases was a key contributor, whereas the production phase accounted for most of the impact in single-use devices. Overall, singleuse fURS had a substantially higher potential environmental and health impact than their reusable counterparts. This study underscores the environmental and health impacts of ureterorenoscopy and highlights the importance of incorporating Planetary Health principles into healthcare practices. It provides a foundation for further analyses and research, aiming to drive transformative action in the healthcare sector toward sustainability.

**Supplementary information:**

The online version contains supplementary material available at 10.1007/s00240-024-01664-2.

## Introduction

Human health depends on a functioning environment [1]. Regrettably, humans are damaging environment through the overuse of finite resources and by pollution [2]. This has both direct effects on public health (e.g., higher rates of asthma in urban areas with high levels of air pollution [3]) and indirect effects on public health (e.g., rising rates of acute kidney failure from heat stroke due to heat waves attributable to global warming [4, 5]). In summary, climate change appears to drive a wide range of growing public health problems [6]. *Planetary Health*, thus, is the basis for human health.

Hospitals are also contributing to environmental damage and consequently impact public and planetary health [7, 8]. Globally, the healthcare sector is responsible for around 4% of greenhouse gas emissions [9]. In 2018, the impact of the US healthcare sector resulted in 553 million metric tons CO_2_ equivalents (CO_2_eq), according to 8.5% of the total domestic US greenhouse gas emissions. The US’ healthcare impacts were estimated to be responsible for a loss of 388.000 DALYs in 2018 [10]. The production and disposal of materials for daily clinical use account for about 19% of the emissions of the healthcare system. Therefore, the selection and handling of medical material can be an important parameter to lessen the climate impact of the healthcare sector [11]. Here, part of the hospital waste is generated by the growing use of single-use products due to ease of use and availability, many of which have reusable versions that could be used instead. In general, the reusable version of a medical instrument is considered to have a lower environmental impact than its single-use equivalent [12].

One such medical device is the flexible ureterorenoscope (fURS). The fURS is commonly used to diagnose and treat large or obstructing kidney stones [13]. It consists of a handle, a flexible tube, and a digital camera for image transmission. The tube contains the working channels for irrigation and insertion of various devices (e.g., wire baskets, electrocautery, biopsy forceps, laser fibers).

fURS are available as single-use or reusable devices, and both types are widely used all over the world for kidney stone surgery [14, 15]. Earlier studies comparing the clinical performance of single-use versus reusable fURS have found no significant differences between them, including for overall success rates, stone-free rates, operating time, and radiation exposure [13, 15, 16]. The environmental and public health impact has received little attention though [17].

Life Cycle Assessment (LCA) is a method to evaluate the potential impact of products considering their entire lifespan, including manufacturing, use, reprocessing/maintenance, and disposal^12^. While it is commonly applied to assess the environmental impact of products, it has just recently been introduced into healthcare research [18, 19]. Most healthcare LCAs have reported only the intermediate outcome of the potential environmental impact based on calculations of CO_2_-emissions, but not the ultimate outcome of the potential public health impact. Moreover, detailed data on individual and adjustable intra-hospital factors, e.g., different energy sources, have not been integrated into the analyses thus far.

In the present analysis, we looked at both, the comparison of CO_2_-emissions, including adjustable factors and scenarios and the resulting potential impacts to human health associated with the various inputs and outputs from the product system of single-use versus reusable fURS. We have used the well-established LCA method. The input data are based on the fURS used at the university medical center Tübingen, Germany.

Finally, with this approach we aim to provide an exemplary illustration of how LCA analysis can be used to assess the potential health impact of reusable vs. single-use medical supplies for future decision making in purchasing of medical material.

## Methods

### Ethics

This study made no use of human subjects or individual patient data and thus is exempt from seeking approval of an institutional review board. No funding was received, directly or indirectly, from any commercial or for-profit entity. It was not possible to involve patients or the public in the design, or conduct, or reporting, or dissemination plans of our research.

### Study design and overview

This study assesses the potential human public-health impact from climate change of processes included in one usage of reusable versus single-use fURS. The analysis had two stages. First, we performed a single-center life cycle assessment (LCA), according to internationally accepted methods (ISO 14040/44) [20–22] to estimate the Global Warming Potential (midpoint) and the potential public health impact (endpoint) of the inputs and outputs from the product system of single-use versus reusable fURS due to climate change. Second, we performed sensitivity analyses to assess how the results change when the input variables and/or modeling assumptions change.

### Life cycle assessment framework

Life cycle assessments evaluate potential impacts to the environment and human health across the entire life cycle of a product or service [20, 21]. The scope of this study was to assess and compare the potential human public health impact of single-use and reusable ureterorenoscopes used at the university medical center the University Hospital of Tübingen regarding climate change aspects. Given that studies have shown that both devices have identical clinical effectiveness and risk for the patients, our unit of analysis for the LCA, the “functional unit”, was “per one use” for each device. The duration of one use was assumed to be one hour.

### Data collection

Data was collected, from 2020 to 2023, about the life-cycle stages (manufacturing, delivery, use, reprocessing/maintenance, disposal) of single-use and reusable fURS. Data were prioritized according to the following hierarchy: (1) direct statements from the companies or staff involved, (2) empirical measurements (e.g., weighing the items) by the investigators, (3) data from the literature, (4) estimates based on expert opinions. Direct statements from companies and staff were obtained through interviews (conducted on-site, online, or by telephone), email correspondence, and written survey questionnaires. Manufacturing companies were contacted for data on the production of fURS, and other companies were contacted as needed for information relevant to the other phases of the life-cycle.

### Estimations and assumptions

Electricity for manufacturing is highly dependent on the location. For single-use fURS made in China, we used Chinese electricity market data. The estimated value on the amount of electricity was confirmed by another manufacturing company. For reusable fURS made in Germany, the conventional German electricity mix listed in the ecoinvent database (ecoinvent version 3.8; Zurich, Switzerland [23]) was used.

For instance, water impacts also depend on the location. For single-use production abroad, background data for “tap water (Rest-of-the-World)” was used. For reusable fURS, European Union tap water was included in the calculation. Sensitivity analyses confirmed that even wildly implausible higher estimates of water usage had no noticeable effect on the results.

External emission parameters linked to ureteroscopy (e.g., anesthetic gases or electricity needed for air conditioning or heating the operating room) were not included in the LCA, as they were assumed to be identical for reusable and singleuse devices. We focused exclusively on the fURS itself.

Reprocessing of reusable fURS takes place at the institutional sterilization unit, a facility certified according to DIN EN ISO 13,485. We proposed data from the literature [17] to the reprocessing managers, who confirmed it as an estimate. The manufacturers of the washing machines (INNOVA E3s CMS DC GL washing and STERRAD^®^ 100NX ALLClear sterilization) used in reprocessing were unable to provide data, so we assumed 9.2 kW/cycle (equivalent to 7.89 kWh), as also reported in a previous study for the Olympus ETD4 sterilization machine [17]. At the university medical center of Tübingen, reprocessing is performed using electricity from 100% renewable sources.

Reusable fURS require periodic maintenance, which is performed at the manufacturing company. We assumed the packaging for maintenance to be similar to the packaging for reprocessing. We assumed transportation to be similar to the transportation to and from the production company to the hospital. We added to this the impact of one reprocessing instance (packaging excluded because already included separately).

For reusable fURS reprocessing, we assumed two devices per washing machine and sterilization process. Although non-standard reusing of *single-use* devices has been researched on in other countries [24], we didn’t consider reusing single-use devices in our study.

When the devices are no longer usable, they are disposed of in accordance with hospital standards. Ureterorenoscopes are classified as waste code 18 01 04 [25], which designates waste for which no special requirements apply to collection and disposal. All waste treated under this code is incinerated at the local residual waste plant. Corresponding incineration processes from ecoinvent cut-off were taken to account for the impacts from disposal. To date, fURS are disposed as waste without recycling, and we assessed that a recycling option wouldn’t change the results noticeably.

### Health impact assessment

The data collected served as the basis for estimating the potential health impacts due to climate change per *one use* of the devices, using the ecoinvent life cycle database, applying the midpoint impact assessment ‘climate change’ method as well as the mid-to-endpoint factors for human health-related climate change impacts of ReCiPe2016 [26]. The potential human health impacts are reported in disability-adjusted life-years (DALY) and were quantified based on the midpoint results as described for ReCiPe2016 (referring to De Schryver et al. (2009)) [27], considering increased risk of health damages due to climate change (1 malnutrition, malaria, diarrhoea and flood risk).

DALYs are the key measurement unit for assessing public health, combining years of life lost to early mortality with the portion of life-time lost due to living in variable states of disability from various illness [28–30]. In LCA, DALYs are used to standardize the potential impacts of environmental burdens.

For example, as part of the reprocessing of reusable fURS, a 20 ml Luer Solo Inject Syringe is used to flush the scope. The package of this syringe weighs approximately 1.28 g and consists mainly of plastic. One syringe is used per fURS. The material was matched with the reference “*market for extrusion*, *plastic film| extrusion*, *plastic film| Cutoff*, *U– GLO*” (U = Unit process; GLO = global) in ecoinvent. Results were added to the calculation.

### Sensitivity analyses

LCA results are subject to assumptions and estimations. To test the robustness of the results, we performed sensitivity analyses on the parameters that were likely to affect the final results. These included four scenarios: (1) different production countries (and thus different energy sources) for the single-use fURS, (2) a different energy mix for use of the fURS at the hospital, (3) a different number of uses of the reusable fURS during its lifetime, (4) a different frequency of repairs of the reusable fURS. The variables of these sensitivity analysis are described further in the Results.

## Results

### Lifespan of a reusable fURS

According to hospital administration data: (a) 160 flexible ureteroscopy procedures are performed per year with reusable devices, (b) 6 reusable fURS are in concurrent use at the hospital, (c) one reusable fURS has an average lifespan of 5 years, and thus we calculated that, on average, a reusable fURS is used 133 times at our hospital from purchase to disposal. Administration reported that maintenance was necessary after every 11 uses on average.

### Material and energy consumption of fURS

An overview of the basic data collected about the material and energy consumption for fURS can be found in Supplemental Tables 1 and more in-depth information about the materials and energy consumed for the reprocessing of reusable fURS can be found in Supplemental appendix 1.

### Health Impact of single-use versus reusable fURS

The potential public health impact of the life cycle of fURS is about four times higher when single-use fURS are used versus reusable fURS: 4.57E-06 vs. 1.15E-06 DALYs per use (4.93 vs. 1.24 kg CO_2_eq).

**Fig. 1:**
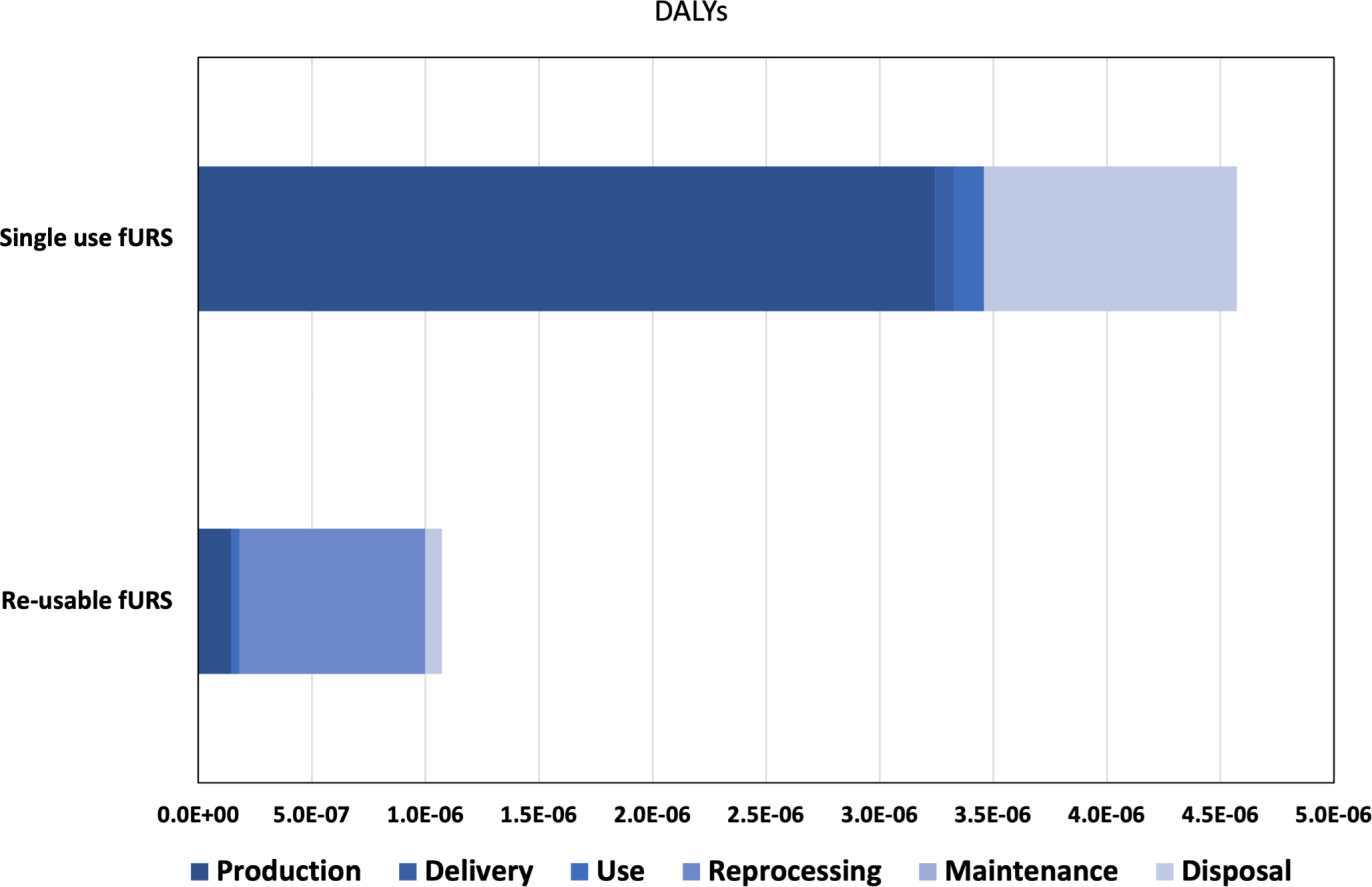
Contributions of the various life cycle stages to the potential health impact of single-use vs. reusable fURS. (Reprocessing and maintenance are not applicable to single-use fURS. Delivery is minuscule for reusable fURS.)

This difference is due almost entirely to the substantial initial health impact for production of each fURS, which is then spread out over multiple uses of a reusable fURS (133 uses in our study) but is repeated for every use of a single-use fURS (Table 1; Fig. 1). The combined health impact from reprocessing, maintenance, and disposal of a reusable fURS was roughly equivalent to the health impact from disposal of a single-use fURS. The health impact from delivery and use of fURS, although much larger for single-use fURS than reusable fURS, was only a small fraction of the overall potential health impact in both cases. Supplemental Table 2 provides a detailed break-down of the results.

**Table 1:** Global Warming Potential (in kg CO_2_eq) and potential health impact (in DALYs) for single-use versus reusable fURS, on a per use basis

Life-Cycle	Single-use fURS		Reusable fURS	
	per use			
Stage	kg CO_2_ eq	DALYs	kg CO_2_ eq	DALYs
Production	3.5E + 00	3.2E-06	1.6E-01	1.4E-07
Delivery	9.2E-02	8.5E-08	8.8E-05	8.2E-11
Use	1.4E-01	1.3E-07	4.0E-02	3.7E-08
Reprocessing	not applicable	not applicable	8.8E-01	8.2E-07
Maintenance	not applicable	not applicable	8.3E-02	8.5E-08
Disposal	1.2E + 00	1.1E-06	7.6E-02	7.1E-08
TOTAL	4.9E + 00	4.6E-06	1.2E + 00	1.2E-06

### Sensitivity analyses: country of production of single-use fURS

The investigated single-use fURS are produced mainly in China but can be purchased from other suppliers elsewhere. For sensitivity analyses, we considered two other manufacturing countries– Malaysia and Germany– to compare other scenarios with different electricity mixes. We note though that Germany does not currently manufacture single-use fURS; this is a hypothetical analysis for the sake of comparability to the reusable fURS.

Our standard baseline scenario of single-use fURS manufacturing in China results in 4.6E-06 DALYs (4.9 kg CO_2_eq) for the whole life cycle. Changing the production site to Malaysia lowers the potential public health impact to 4.1E-06 DALYs (4.41 kg CO_2_eq). A hypothetical scenario of single-use fURS manufactured in Germany would lower the potential health impacts to 3.4E-06 DALYs (3.6 kg CO_2_eq).

### Sensitivity analyses: Energy Mix at the hospital

Our hospital uses energy from 100% renewable sources. For sensitivity analysis, we compared a scenario with the conventional energy mix in Germany (including nuclear, coal, natural gas, other fossil fuels, and renewable sources (based on the underlying ecoinvent 3.8 dataset).

**Fig. 2:**
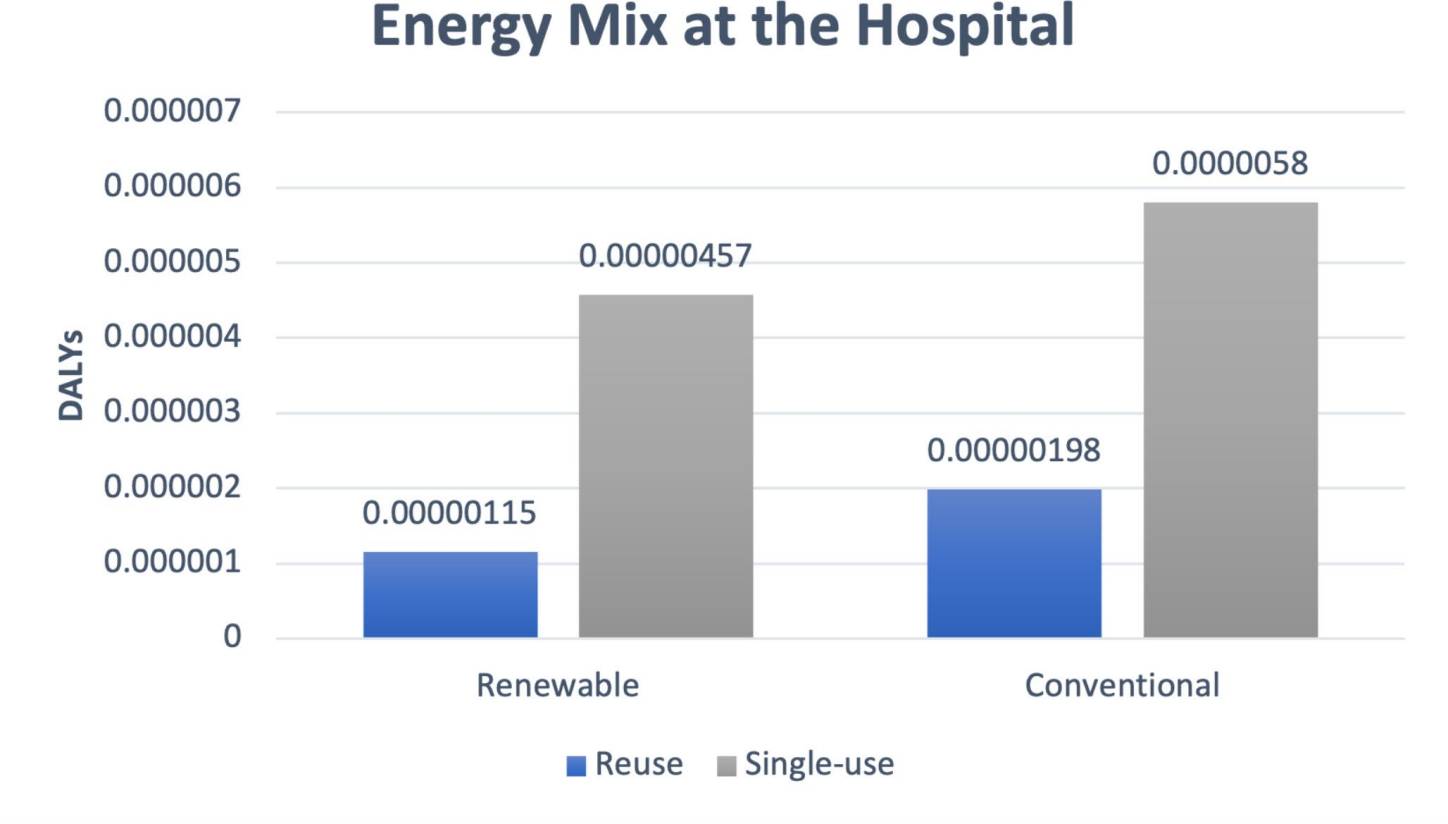
Sensitivity analysis: Renewable vs. conventional energy mix

If a conventional energy mix is used at the hospital, the health impact in our study is 4.01E-06 DALYs (4.32 kg CO_2_eq) for the reusable fURS versus 5.8E-06 DALYs (6.25 kg CO_2_eq) for the single-use device. This is higher for both types of fURS as compared to renewable sources, and indeed the increase is greater for reusable fURS than it is for single-use fURS. (Fig. 2**)**

### Sensitivity analyses: number of Lifetime uses of one reusable fURS

According to data from the hospital administration, we estimated that each reusable fURS is used on average 133 times. This number is depending on the type of use/ surgery, the experience of the surgeon and material wear. Variable estimates were published in earlier studies. Since an Australian study estimated that fURS were reused 180 times at their hospital [17], we performed a sensitivity analysis with this higher number of uses. Here, the potential public health impact is reduced from 1.15E-06 (1.24 kg CO_2_eq) to 1.1E-06 DALYs (1.17 kg CO_2_eq).

**Fig. 3:**
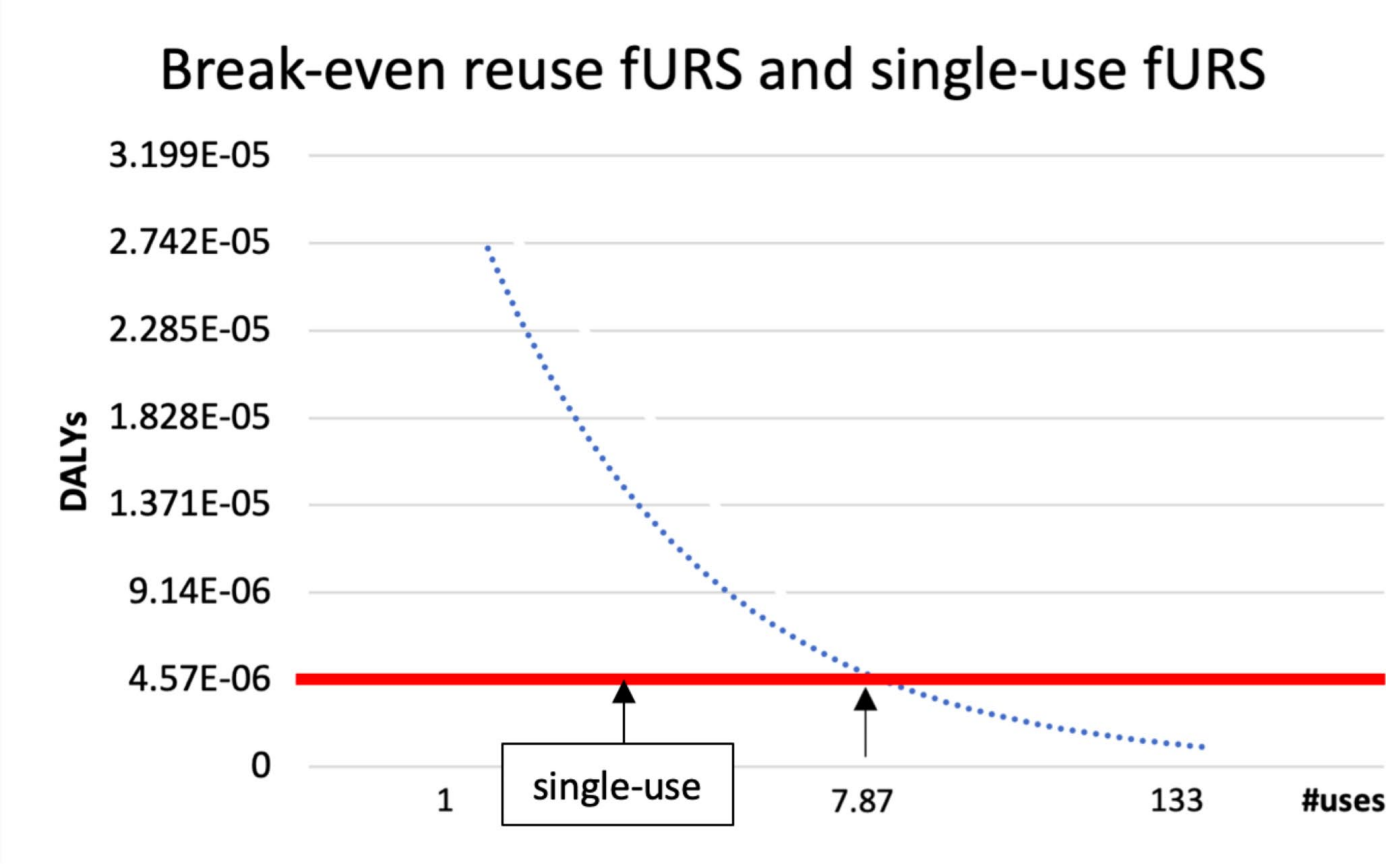
Break-even point: After approximately eight uses the potential public health impact of single-use and reusable fURS was equal

In a separate “break-even” analysis, we found that the potential public health impact of single-use and reusable fURS was the same when reusable fURS were used 7.9 times (Fig. 3). In other words, a mere 8 uses of a reusable fURS suffices for reusable fURS to have less public health impact than single-use fURS.

### Sensitivity analyses: frequency of repairs of a reusable fURS

**Fig. 4:**
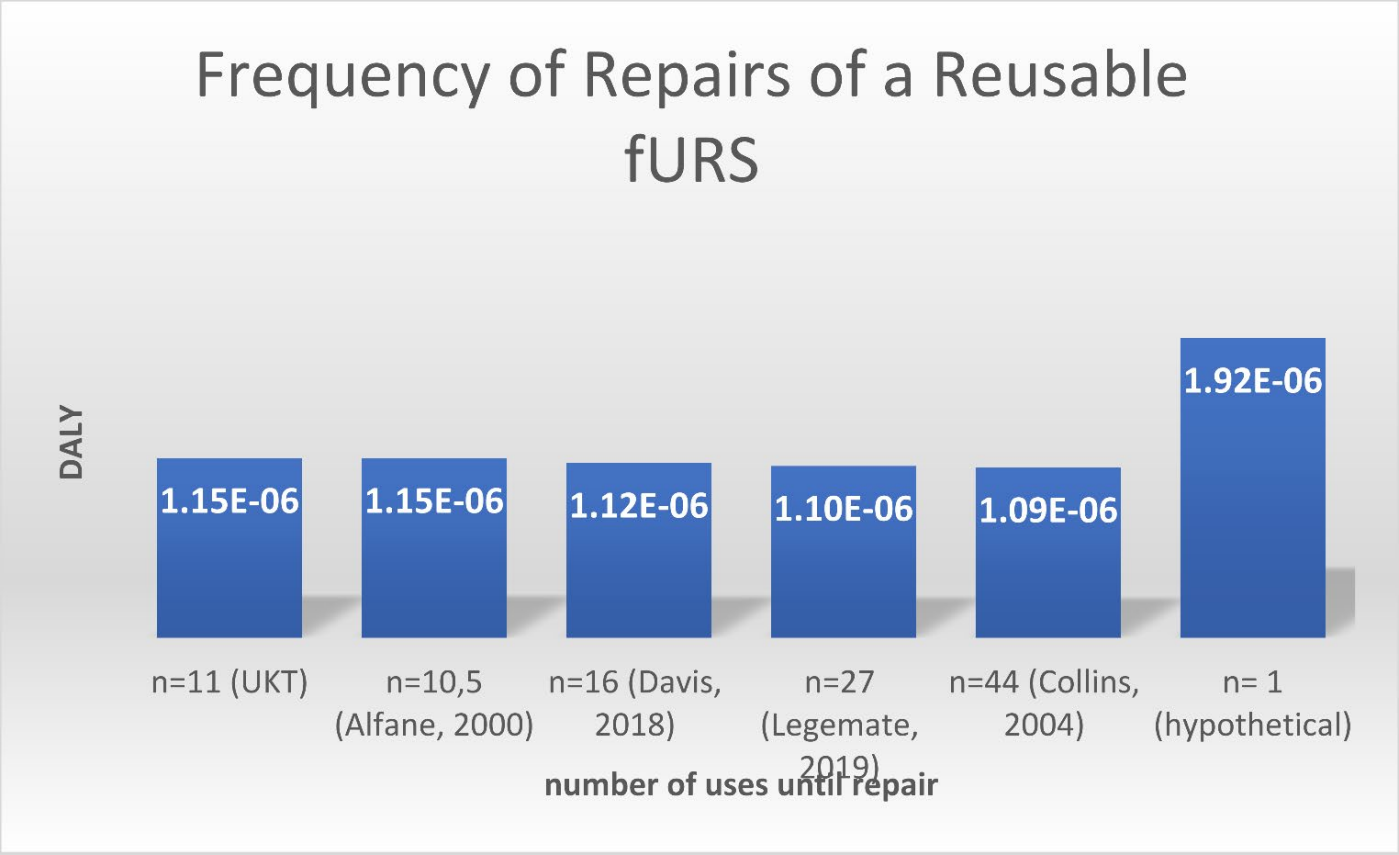
DALYs resulting from different numbers of uses until repair

According to institutional data, each reusable fURS is repaired after 11 uses on average. Our estimate was supported by one previous study, which estimated that reusable fURS are repaired after every 6–15 uses [31]. Other studies have estimated that reusable fURS are repaired only after every 16th use [17] or every 27th procedure [32], so we performed sensitivity analyses with those less frequent rates of repair. Decreased frequency of repair had only marginal effect: if maintenance takes place after 27 uses, the potential public health impact is still 1.1E-06 DALYs (1.19 kg CO_2_eq). An increase of repairs or repair after each use (1.92E-06 DALYs, 2.07 kg CO_2_eq)) would still not break-even. (Fig. 4**)**

## Discussion

The results of this LCA provide three basic insights. First, the potential health impact is substantially higher when using single-use fURS than when using reusable fURS in the investigated scenario (4.57E-06 vs. 1.15E-06 DALYs per use). Second, this difference is due almost entirely to the substantial initial impact for production of a fURS. The potential impact from the rest of the lifecycle altogether (delivery, use, reprocessing, maintenance, and disposal) is similar for both devices (1.3E-06 DALYs for single-use versus 1.0E-0.6 DALYs for reusable fURS). Third, after making that initial purchasing choice, there seem to be limited options to overcome the fundamental difference between single-use and reusable fURS. The sensitivity analyses showed that changing the country of the supplier, the frequency of reuse or maintenance, the type of energy sources used, will not tip the scales in favor of single-use fURS so long as reuable fURS are used on average at least 8 times– a very low threshold of reuse. However, decisions about suppliers, energy, frequency of reuse, etc. are important factors influencing the global health impact of reusable fURS itself, since the analysis revealed that energy sources used for refurbishment and sterilization are relevant contributive factors here.

Davis et al. previously compared reusable vs. single-use fURS, but they concluded that there was no difference in the environmental impact [17]. However, total results for reusable fURS (4.47 kg CO_2_ ≈ 4.14E-06 DALYs) were notably higher than in our investigation (1.24 kg CO_2_eq ≈ 1.15E-0.6 DALYs) due to a high impact for reprocessing– probably because of the fact that the study was performed in Australia, where the main source of energy is coal. This delineates energy source as the main contributive factor for the CO_2_ footprint of reusable fURS.

Our study has specific limitations that must be mentioned. (1) The data collected for LCAs are based on estimations, as described in the methods and supplemental files. The resulting– potential– impact is always an indication of a possible effect/damage, not an absolute amount. We tried to counteract this by gathering data from multiple sources, including the literature. In addition, we only included data on the endoscope itself, not gathering information on the surrounding operation room processes. (2) This was a single-center study, results might differ at other hospitals, using different devices, energy sources, and processes. The results were not sensitive to the country of the supplier, the frequency of reuse or maintenance, or the type of energy sources used. Reusable fURS used at least eight times will have fewer impacts than single-use fURS. (3) Our LCA relied essentially on the ecoinvent database to provide information on the CO_2_ emission associated with the used products/energy, etc. Conversion into potential health impacts (DALYs) was done as described in ReCiPe2016, respectively by DeSchryver et al. [27]. Other theoretical health relevant aspects for reusable or single-use fURS may be present, e.g., if any reprocessing staff were ever getting sick from the materials or chemicals they are using, which that would likely override the public health benefit of reusable devices. However, according to the product information for the used substances referring to the European chemicals legislation, and the fact there is no direct skin contact, there is no indication of health hazard caused by reprocessing. After all, nearly 29.000 ureterorenoscopies are performed in Germany every year [33] to lessen public kidney health damage and the patient’s personal pain through urolothiasis.

In closing, we should consider what this difference in the impacts to human health from climate change really means. Admittedly, the impact from fURS at one hospital is small by itself, but hospitals are making similar choices about comparable devices and numbers add up from these choices [34]. To summarize, the present study generated approximate numbers on single-use versus reusable fURS that allow a comparison in Europe in the first place, but we also developed a more general methodological model and conceptual framework for similar analyses of other devices, products, and choices in the healthcare sector and beyond. The substantial long-term (*planetary*) health consequences of global warming are already sufficiently clear. It is urgent that we act now to prevent them.

## Electronic supplementary material

Below is the link to the electronic supplementary material.

Supplementary Material 1

## Data Availability

No datasets were generated or analysed during the current study.
