# Supplementary material for: Potential impacts to human health from climate change: A comparative life-cycle assessment of single-use versus reusable devices flexible ureteroscopes
Source: Urolithiasis. 2024 Nov 23;52(1):166. doi: 10.1007/s00240-024-01664-2 (PMC11585494; doi:10.1007/s00240-024-01664-2)
Supplement: Supplementary file 1 — Supplementary Material 1 [file 240_2024_1664_MOESM1_ESM.docx]

**Supplemental Table 1.** Overview of the data used in the LCA. **A)** Single-use fURS. **B)** Reusable fURS.

**A) Single-use fURS.** (The categories of “Reprocessing” and “Repair” are not applicable for single-use fURS.)

| **Life-Cycle Stage** | **Material or Process** | **Quantity for *one use*** | **Unit** | **Data Type / Source** 1=statements, 2=measures, 3=literature, 4=estimation | **Notes** |
| --- | --- | --- | --- | --- | --- |
| **Production** |  |  |  |  |  |
|  | plastic | 189.25 | g | 1 |  |
|  | metal | 18.55 | g | 1 |  |
|  | electronics | 0.19 | g | 1 |  |
|  | packaging: paper | 62.2 | g | 1 |  |
|  | packaging: cardboard | 110.05 | g | 1 |  |
|  | other (glue) | 0.4 | bottle | 1 |  |
|  | ETO | 8.2 | g | 1 | ethylene oxide sterilization |
|  | water | 111 | L | 1 | water use (production) around 200 metric tonnes/month for 1800 devices/month |
|  | electricity | 2.4 | kWh | 4 |  |
| **Delivery** |  |  |  |  |  |
|  | transportation | 13,000 | km | 4 | transportation by ship/truck (from manufacturer to hospital) |
| **Use** |  |  |  |  |  |
|  | electricity | 2.4 | kWh | 1 | we assume the same amount of energy for use of ru and su fURS |
| **Disposal** |  |  |  |  |  |
|  | packaging,  various materials | 380 | g | 1 | partly recyclable: cardboard and paper |
|  | transportation | 30 | km | 2 | transportation by truck (from hospital to incineration plant) |

**B) Reusable fURS.**

| **Life-Cycle Stage** | **Material or Process** | **Quantity for *one use*** | **Unit** | **Data Type / Source** 1=statements, 2=measures, 3=literature, 4=estimation | **Notes** |
| --- | --- | --- | --- | --- | --- |
| **Production** |  |  |  |  | assuming 133uses |
|  | plastic | 0.75 | g | 4 |  |
|  | metal | 3.01 | g | 4 |  |
|  | electronics | 0.05 | g | 3 |  |
|  | packaging: cardboard & paper | 0.42 | g | 3 |  |
|  | packaging: plastic | 19.68 | g | 3 |  |
|  | water | 0.83 | L | 1 |  |
| **Delivery** |  |  |  |  | assuming 133 uses |
|  |  | 0.69 | km | 2 |  |
| **Use** |  |  |  |  |  |
|  |  | 2.4 | kWh | 1 | we assumed the same amount of energy for use of ru and su fURS |
| **Reprossessing** |  |  |  |  |  |
|  | electricity | 3.95 | kWh | 1,2,3 |  |
|  | manual cleaning: plastic | 64.17 | g | 2 |  |
|  | manual cleaning: water | 28.8 | L | 2 |  |
|  | manual cleaning: cleansing agent | 0.06 | L | 2 |  |
|  | machine cleaning: cleansing agent | 0.0032 | L | 2 | assuming 2 ru fURS / machine / wash cycle |
|  | machine cleaning: water | 24 | L | 2 | assuming 2 ru fURS / machine / wash cycle |
|  | machine cleaning: disinfectant | 6.2 | g | 2 | assuming 2 ru fURS / machine / wash cycle |
|  | machine cleaning: aer medicinalis | 0.0145 | L | 4 | assuming 2 ru fURS / machine / wash cycle |
|  | packaging: plastic | 148 | g | 2 |  |
|  | sterilization: hydrogen peroxide | 8.12 | ml | 2 |  |
|  | sterilization: high voltage current | 0.5 | kWh | 2 |  |
| **Repair** |  |  |  |  | assuming repair after every 11^th^ use |
|  | transportation | 16.55 | km | 2 |  |
|  | water | 0.09 | L | 2 |  |
|  | plastics | 2.93 | g | 2 |  |
| **Disposal** |  |  |  | 2 | assuming 133 uses |
|  | plastic, metal, other | 5.12 | g | 4 |  |
|  | transportation | 0.23 | km | 2 | transportation by truck (from hospital to incineration plant) |

**Table 2.** Detailed Global Warming Potential (in kg CO_2_eq) and Health Impact (in DALYs) of the components of single-use versus reusable fURS, on a per use basis.

| **Life-Cycle Stage**  [components contributing to impact] | **Single-use fURS** | | **Reusable fURS** | |
| --- | --- | --- | --- | --- |
|  | **per use** | | | |
|  | **kg CO_2_eq** | **DALYs** | **kg CO_2_eq** | **DALYs** |
| **PRODUCTION** |  |  |  |  |
| metal | 1.69E-01 | 1.57E-07 | 1.34E-02 | 1.24E-08 |
| plastic | 6.46E-01 | 6.00E-07 | 2.70E-03 | 2.50E-09 |
| rubber | - | - | 2.10E-03 | 1.95E-09 |
| glass | - | - | 1.39E-04 | 1.29E-10 |
| electronics | 1.59E-03 | 1.47E-09 | 3,90E-04 | 3.62E-10 |
| glue | 1.64E-02 | 1.53E-08 | - | - |
| packaging | 1.12E-01 | 1.04E-07 | 1.27E-01 | 1.18E-07 |
| electricity | 2.54E+00 | 2.35E-06 | 1.02E-02 | 9.42E-09 |
| water | 1.17E-04 | 1.08E-10 | 2.84E-07 | 2.64E-13 |
| Ethylene oxide (ETO) sterilization | 1.56E-02 | 1.446E.08 | - | - |
| **Production Subtotal** | **3.50E+00** | **3.244E-06** | **1.56E-01** | **1.44E-07** |
|  |  |  |  |  |
| **DELIVERY** |  |  |  |  |
| transportation: ship | 4.74E-02 | 4.40E-08 | - | - |
| transportation: truck | 4.41E-02 | 4.09E-08 | 8.81E-05 | 8.18E-11 |
| **Delivery Subtotal** | **9.15E-02** | **8.49E-08** | **8.81E-05** | **8.18E-11** |
|  |  |  |  |  |
| **USE** |  |  |  |  |
| electricity | 1.40E-01 | 1.30E-07 | 3.98E-02 | 3.70E-08 |
| **Use Subtotal** | **1.40E-01** | **1.30E-07** | **3.98E-02** | **3.70E-08** |
|  |  |  |  |  |
| **REPROCESSING** |  |  |  |  |
| protection foil for inner-clinic transportation | - | - | 1.02E-02 | 9.50E-09 |
| PPE^[[1]](#footnote-1)^ materials | - | - | 5.79E-02 | 5.37E-08 |
| materials for manual pre-cleaning | - | - | 1.43E-01 | 1.33E-07 |
| materials for mechanical pre-cleaning | - | - | 3.27E-03 | 3.06E-09 |
| neutralization (water) | - | - | 2.70E-03 | 2.53E-09 |
| chemical disinfection | - | - | 1.28E02 | 1.19E-08 |
| flushing (water) | - | - | 2.70E-03 | 2.53E-09 |
| drying (medical air) | - | - | 3.43E-03 | 3.18E-09 |
| packaging (“Tyvek”) | - | - | 3.44E-01 | 3.19E-07 |
| sterilization (H_2_0_2_ + electricity) | - | - | 7.83E-02 | 7.27E-08 |
| packaging (LDPE) | - | - | 1.02E-02 | 9.50E-09 |
| electricity | - | - | 2.12E-01 | 1.97E-07 |
| **Reprocessing Subtotal** | **not applicable** | **not applicable** | **8.80E-01** | **8.17E-07** |
|  |  |  |  |  |
| **MAINTENANCE** |  |  |  |  |
| transportation: truck | - | - | 2.13E-03 | 1.98E-09 |
| packaging | - | - | 9.31E-04 | 8.64E-10 |
| reprocessing | - | - | 8.00E-02 | 7.43E-08 |
| **Maintenance Subtotal** | **not applicable** | **not applicable** | **8.31E-02** | **7.71E-08** |
|  |  |  |  |  |
| **DISPOSAL** |  |  |  |  |
| transportation: truck | 1.89E-03 | 1.75E-09 | 1.20E-04 | 1.12E-10 |
| material waste | 1.20E+00 | 1.11E-06 | 7.63E-02 | 7.08E-08 |
| **Disposal Subtotal** | **1.20E+00** | **1.12E-06** | **7.64E-02** | **7.09E-08** |
|  |  |  |  |  |
| **TOTAL** | **4.93E+00** | **4.57E-06** | **1.24E+00** | **1.15E-06** |

**Supplemental Appendix 1. Detailed description of the reprocessing of reusable fURS.**

The reprocessing of reusable fURS consists of the following stages: 1) transportation from the OR to the central sterilization unit (not included in data analysis), 2) use of protective equipment, 3) manual pre-cleaning, 4) mechanical cleaning, 5) neutralization, 6) chemical disinfection, 7) flushing, 8) drying, 9) packing, 10) sterilization, 11) packing, 12) work surface cleaning.

1) fURS are transported from the OR to the central sterilization unit by hospital staff. The impact of this step was considered negligible and is not included in the LCA.

2) The personal protective equipment (PPE) of the sterilization department for the reprocessing of ureteroscopes consists of gowns (waterproof, long-sleeved), gloves (long-shouldered), reusable customizable protective shield or goggles (depending on individual staff preferences), and mouth-nose protection (disposable). We weighed these various items individually.

3) Manual pre-cleaning is performed with 1.2L EndoCLEAN (Dr. Weigert neodisher endo CLEAN). This is a solvent-based washing and cleaning agent produced for flexible endoscopes in washer-disinfectors (RDG-E). It contains sodium cumenesulfonate (≥ 1% and <10%) CAS No. 15763-76-5 and fatty alcohols, alkoxylated (<1%). Thus, 0.06 L sodium cumenesulfonate and 0.012 L fatty alcohols were included for the calculation of the LCA. The devices are pre-cleaned with the aid of a fine brush, which was assumed to be made of polypropylene (PP), as it is made entirely of plastic. It weighs approximately 1.5 g and is a single use product. The cloth for pre-cleaning is made of fleece and weighs 21 g. Manual pre-cleaning requires 28.8 L water/device. Connection to power for leakage testing (done several times during the reprocessing process) is done manually and the power consumption is negligible. A disposable syringe (20 ml Luer Solo Injekt; Braun; Melsungen, Germany) is used for flushing at the end of this reprocessing step, weight: approx. 10 g. The packaging of this syringe (1.28g in total; plastic and paper) is included in the calculation as plastic (polyethylene, PE) due to its predominant proportion.

4) From the step of machine cleaning onwards, two devices per cycle are assumed, since the machines hold two devices. This assumption corresponds to the optimum capacity utilization during reprocessing. Therefore, the following reprocessing steps (machine cleaning, neutralization, chemical disinfection, drying, sterilization) are each multiplied by a factor of ½, since we have defined the life cycle of one use of one fURS as an impact category. The cleaning agent Dr. Weigert neodisher endoCLEAN) is also used for machine cleaning, albeit only 59 ml. This results in approximately 3.2 ml (0.0032 L) and less than 0.59 ml (<0.00059 L) for the calculation of the life cycle assessment of sodium cumenesulfonate and fatty alcohols. For the washing device (INNOVA E3s CMS DC GL at the Otfried-Müller-Straße 4 site) and the standard reprocessing program (“Endo-Normal-DC-GL”), fully demineralized water is used (11.8 L per 2 devices).

5) Twelve liters of water are used in the neutralization process.

6) Chemical disinfection is performed by Dr. Weigert neodisher endoSEPT 1.0%, which is matched to the cleaning agent used. Thus, carryover of the agent from the cleaning liquid into the disinfection step does not impair the disinfection performance, according to the manufacturer (Chemische Fabrik Dr. Weigert GmbH & Co. KG; Hamburg, Germany; https://www.drweigert.com/de, accessed on 6 January 2023). The antiseptic effect of the machine-applied disinfectant is based on the aldehyde glutaraldehyde it contains. With a dosage of 118 ml to 11.8 L water, 12.4 g glutaraldehyde can be calculated (0.118 L neodisher endoSEPT with 10.5g glutaraldehyde in 100g means 12.4g glutaraldehyde in 118g neodisher endo®SEPT).

7) This is followed by flushing with 12 L of water and another leakage test. As described above, leak tests are carried out at various points in the preparation process using a manually operated pump similar to a blood pressure measuring pump. App. 14.5 ml of medical compressed air, aer medicinalis (ATC code: V03AN05), is used for this purpose (rough estimation: Every 3 weeks a change of gas bottle (10L bottle) results in 10L per 15 working days or 0.7L per working day. One fURS every other day: 0.7 / 2 = 0.35. Processing approx. 1h: 0.35L / 24h = 0.0146L = 14.5ml), this being a hypothetical 24h reprocessing. Other equipment for leakage testing is frequently used and therefore not included.

8) Aer medicinalis is also used to dry the equipment after flushing (amount negligible). This is supplied to the hospital in gas cylinders (steel), (replaced approximately every three weeks. (Reusable) Gas cylinders not included).

9) Due to the subsequent sterilization, special packaging of the fURS is then performed using Tyvek®. For packaging, 120 cm x 120 cm are needed per fURS. The weight of this material is approx. 144 g per fURS.

10) Sterilization takes place using hydrogen peroxide (58%), which is upregulated to 98%. The energy consumption figure for this was requested by the sterilization management from the medical technology company MMM Münchener Medizintechnik Mechanik GmbH and can be gathered from the installation requirements of the device (STERRAD). Germs are eliminated by the reaction of O^-^, which is produced during the reaction of H_2_O_2_ to H_2_ and O^-^. H_2_O und O_2_ result as “waste products”. A more in-depth inquiry led to an amount of 10.8 ml H_2_O_2_: two 58% ampoules of hydrogen peroxide of 7ml each with 5400 microliter H_2_O_2_ / sterilization process result in 10,800 microliters of H_2_O_2_ per sterilization, so 10.8ml were included in the data. High voltage is needed for the upregulation of the hydrogen peroxide ampoules for the sterilization of flexible URS. This process requires 1.41 kWh assuming two devices per process). Two fURS can be sterilized at the same time. In 2/3 of cases, two fURS are sterilized in parallel in the STERRAD device at the UKT. In one third of cases, only one fURS is sterilized singularly. Thus, we took 2/3 as the factor for the input data included in the sterilization (amount of H_2_O_2_ and connection to high current).

11) Finally, a dust cover is applied so that sterile goods remain sterile. Low-density polyethylene is assumed as the material (approx. 4 g).

12) For the cleaning of the working surfaces, a further cloth of 21 g is assumed. But this material was omitted from the further analysis, because this cleaning of the working surfaces is needed due to the reprocessing of numerous other instruments on that same work surface besides the fURS, so the contribution of the fURS to this cleaning need is minimal.

1. Personal protective equipment [↑](#footnote-ref-1)
